# Artificial neural network models for prediction of intestinal permeability of oligopeptides

**DOI:** 10.1186/1471-2105-8-245

**Published:** 2007-07-11

**Authors:** Eunkyoung Jung, Junhyoung Kim, Minkyoung Kim, Dong Hyun Jung, Hokyoung Rhee, Jae-Min Shin, Kihang Choi, Sang-Kee Kang, Min-Kook Kim, Cheol-Heui Yun, Yun-Jaie Choi, Seung-Hoon Choi

**Affiliations:** 1 Insilicotech Co. Ltd., A-1101 Kolontripolis, 210, Geumgok-Dong, Bundang-Gu, Seongnam-Shi, 463-943, Korea; 2 SBScience Co. Ltd., Sung-Ok BD, Sunae-Dong, Bundang-Gu, Seongnam-Shi, 463-825, Korea; 3 Department of Chemistry, Korea University, 1, Anam-dong 5-Ga, Seongbuk-Gu, Seoul, 136-701, Korea; 4 School of Agriculture Biotechnology, Seoul National University, San56-1, Shilim-Dong, Kwanak-gu, 151-742, Korea

## Abstract

**Background:**

Oral delivery is a highly desirable property for candidate drugs under development. Computational modeling could provide a quick and inexpensive way to assess the intestinal permeability of a molecule. Although there have been several studies aimed at predicting the intestinal absorption of chemical compounds, there have been no attempts to predict intestinal permeability on the basis of peptide sequence information. To develop models for predicting the intestinal permeability of peptides, we adopted an artificial neural network as a machine-learning algorithm. The positive control data consisted of intestinal barrier-permeable peptides obtained by the peroral phage display technique, and the negative control data were prepared from random sequences.

**Results:**

The capacity of our models to make appropriate predictions was validated by statistical indicators including sensitivity, specificity, enrichment curve, and the area under the receiver operating characteristic (ROC) curve (the ROC score). The training and test set statistics indicated that our models were of strikingly good quality and could discriminate between permeable and random sequences with a high level of confidence.

**Conclusion:**

We developed artificial neural network models to predict the intestinal permeabilities of oligopeptides on the basis of peptide sequence information. Both binary and VHSE (principal components score Vectors of Hydrophobic, Steric and Electronic properties) descriptors produced statistically significant training models; the models with simple neural network architectures showed slightly greater predictive power than those with complex ones. We anticipate that our models will be applicable to the selection of intestinal barrier-permeable peptides for generating peptide drugs or peptidomimetics.

## Background

Successful drug development requires not only the optimization of pharmacological specificity and potency, but also a method for efficient drug delivery to the target site. Many drug candidates fail to achieve their therapeutic potentials because of poor bioavailability [1]. Oral drug delivery avoids the pain and discomfort associated with injections and also the risk of accidents and infections caused by misuse of needles. For these reasons, the oral route is by far the easiest and most convenient mode of drug administration, and oral availability is a highly desirable property for candidate drugs under development. However, before an orally administered drug can reach its site of action, it must first cross the intestinal epithelial barrier by passive diffusion, carrier- or receptor-mediated uptake or active transport and enter the systemic circulation [2]. Molecules with low permeability and/or absorption rates are not suitable for oral administration, and there has been great interest in finding ways to avoid producing potent but non-permeating molecules [3]. Several screening paradigms for evaluating drug absorption have been employed to enhance the probability of success through the stages of drug development and a number of methods have been developed to assess oral availability using *in vivo*, *in vitro*, *in situ *or *in silico *models [4].

The most widely-accepted *in vitro *absorption model uses Caco-2 cell monolayers. Because Caco-2 cells express several types of transporter proteins, both the passive and active transport potentials of a compound can be investigated [5-7] and several experimental methods have been developed using this model to test the absorption of drugs by the human intestine [8-10]. However, these experimental cell-system methods are rather labor-intensive and not easily applicable to high-throughput screening. As an alternative approach, computational modeling can provide a quick and inexpensive way of evaluating the intestinal permeability of a compound before synthesis. This enables us to prioritize molecules for *in vitro *and *in vivo *studies and improve the overall properties of the compounds that proceed along the drug discovery pathway. A number of models for Caco-2 cell permeability or human intestinal absorption have been reported that predict the oral absorption properties of drugs, mostly limited to small organic molecules [11-14].

Rapid developments in biotechnology and peptide synthesis have made it possible to exploit the unique pharmacological activities of peptides; thousands of different peptides have been designed, synthesized and subjected to a range of screening procedures and biological assays. To analyze the vast amounts of biological data on peptides, quantitative structure-activity relationship (QSAR) models have been successfully employed. For example, several QSAR models have been developed to predict the peptide binding activities of target proteins, resulting in good correlations with *in vitro *data [15-19], and these have proved useful in generating leads through the screening of large peptide libraries. It is surprising that QSAR models have seldom been applied to other pharmacological properties of peptides, especially since failure to comply with pharmacological demands is likely to terminate the development of a candidate peptide drug [20,21]. Although a few previous QSAR studies have investigated the affinities of peptides to intestinal transport proteins, the machine-learning processes were performed not on the basis of sequence information but of chemical structure [22-24]. There have been a few reports on the prediction of intestinal absorption of non-peptide compounds from molecular structure. Wessel et al. reported a QSAR study on a set of 86 compounds with known percentage human intestinal absorption (%HIA) values [25]. To obtain a predictive model, they used a neural network to map molecular structure descriptors to %HIA. Polley et al. applied Bayesian regularized neural networks to develop a statistically significant QSAR model for human intestinal absorption [26].

In this work, we report the first QSAR models to predict the intestinal permeabilities of peptides on the basis of their sequences. A group of peptides crossing the intestinal barrier were selected from a random phage-peptide library using the 'peroral phage display technique', a newly developed *in vivo *technique in which a phage-peptide library is administered orally to rats and the intestinal barrier-permeable phages are collected from the internal organs. Using the sequence set of the selected phage-displayed peptides, we constructed an artificial neural network model to evaluate the intestinal permeabilities of peptides using various descriptors of the physicochemical properties and occurrence of the amino acid residues.

## Results

Using the peroral phage display technique, we identified 852 heptapeptide sequences from phages randomly selected from 10^5 ^~10^7 ^clones translocated from the intestinal lumen to the inner organs such as liver, lung, spleen and kidney (see the Methods section for details). These intestinal barrier-permeable peptides were used as the positive control set for further analysis. Because the phospholipid bilayer is the structural basis of cellular membranes, both hydrophilic and hydrophobic interactions might affect the intestinal permeability of a molecule. To evaluate the effects of individual amino acid residues on the intestinal permeability of a peptide, we compared the frequencies of occurrence of each residue in the intestinal barrier-permeable peptides and in the random phage library (Table 1), then investigated the correlation between the relative residue frequencies and their hydrophobic and hydrophilic properties [27]. We found no direct relationship between the relative frequency and any of the hydrophobicity or hydrophilicity indices investigated (Table 1). Obviously, the intestinal permeability of a peptide is not predictable simply from its hydrophobicity/hydrophilicity, so we proceeded to develop an artificial neural network model that also takes account of the peptide sequence in predicting permeability.

**Table 1:** Comparison for relative hydrophilicity and hydrophobicity of amino acids for the real data sets.

				Hydrophobicity*				
					
Amino acid	Homing^a^	Random^b^	Ratio^c^	Calculated^d^	Side-chain analogues^e^	Amino acids^f^	N-acetyl amides^g^	Hydrophilicity*
Alanine	6.85	6.50	1.05	-0.39	-0.87	-0.50	-0.31	-0.45
Glycine	4.02	2.20	1.83	0.00	0.00	0.00	0.00	0.00
Isoleucine	1.56	2.10	0.74	-1.82	-3.98	-1.80	-1.80	-0.24
Leucine	7.19	9.60	0.75	-1.82	-3.98	-1.80	-1.70	-0.11
Valine	1.98	1.90	1.04	-1.30	-3.10	-1.50	-1.22	-0.40
Methionine	2.69	3.30	0.82	-0.96	-1.41	-1.30	-1.23	-3.87
Phenylalanine	1.56	2.10	0.74	-2.27	-2.04	-2.50	-1.79	-3.15
Tryptophan	0.66	1.90	0.35	-2.13	-1.39	-3.40	-2.25	-8.27
Proline	11.41	10.70	1.07	-0.99	-	-1.40	-0.72	-
Cysteine	0.02	0.00	-	-0.99	-0.34	-1.00	-1.54	-3.63
Serine	13.42	8.60	1.56	1.24	4.34	0.30	0.04	-7.45
Threonine	10.72	13.10	0.82	1.00	3.51	-0.40	-0.26	-7.27
Tyrosine	1.95	2.40	0.81	-1.47	1.08	-2.30	-0.96	-8.50
Asparagine	6.22	6.40	0.97	1.91	7.58	0.20	0.60	-12.07
Glutamine	7.56	7.10	1.06	1.30	6.48	0.20	0.22	-11.77
Histidine	6.74	6.90	0.98	0.64	5.60	-0.50	-0.13	-12.66
Lysine	5.33	3.80	1.40	2.77	6.49	3.00	0.99	-11.91
Arginine	4.89	4.30	1.14	3.95	15.86	3.00	1.01	-22.31
Aspartic acid	3.06	4.10	0.75	3.81	9.66	2.50	0.77	-13.34
Glutamic acid	2.18	3.10	0.70	2.91	7.75	2.50	0.64	-12.63

Correlation Coefficient^h^				0.17	0.21	0.40	0.50	0.03

^a ^Observed frequency of each amino acid in the tissue-homing heptapeptide set obtained from peroral phage display

^b ^Observed frequency of each amino acid in the phage library (Ph. D-C7C™ library)

^c ^Relative ratio of amino acid frequency in homing peptide^a ^to amino acid frequency in phage library^b^

^d ^Calculated from hydrophobicities of the individual groups that make up each side chain, using data for the partition coefficient between water and octanol of many model compounds.

^e ^Hydrophilicity was measured by the partition coefficient *K*_*D *_of the model for each side chain from vapor → water; hydrophobicity for water → cyclohexane. For ionizing side chains, the values were corrected for the fraction of each side chain that is ionized at pH 7. Both scales were normalized to zero for the value of Gly.

^f ^Some values were measured from the relative solubilities of the amino acid in water and ethanol or dioxane.

^g ^Measured from the partition coefficient between water and octanol of the N-acetyl amino acid amides.

^h ^Correlation coefficients between relative ratio^c ^and each hydrophobicity/hydrophilicity.

*Reference [26].

**Figure 1:**
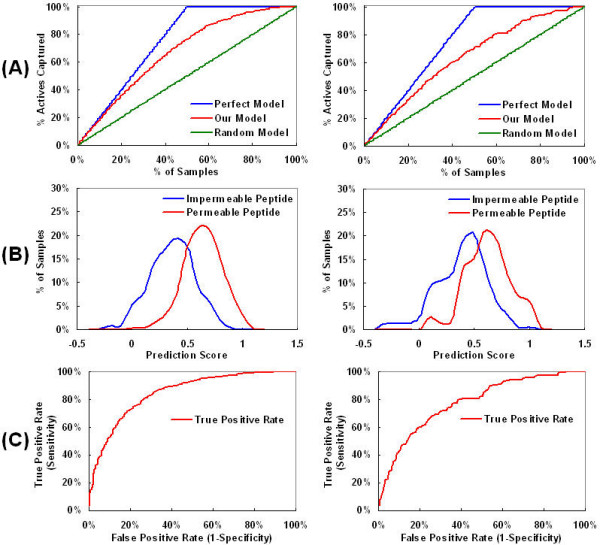
**Predictive features of the model**. The model was constructed with zero neuron in a hidden layer and one in an output layer using binary descriptors. (A) Enrichment curve, (B) Histogram Actives *vs*. Model values, and (C) Receiver Operating Characteristic (ROC) curve. The features for the training and test set were plotted in the left and right panels, respectively.

First, 852 random heptapeptide sequences were generated as negative control data, keeping the frequency of each amino acid residue the same as in the random phage library. We utilized a feed-forward neural network for our sequence-based permeability prediction. Eight models were derived for training data set by varying the type of peptide descriptor and/or the number of neurons in the (single) hidden layer. The predictive features of the resulting model are illustrated in Figure 1, which clearly shows that our model can distinguish effectively between intestinal barrier-permeable and impermeable peptides.

Tables 2 shows the accuracy of prediction by our models for 1:1 data set using binary and VHSE (principal components score Vectors of Hydrophobic, Steric and Electronic properties) descriptors, respectively (see the Methods section for details). The receiver operating characteristics (ROC) score, which is the area under the ROC curve, was used as the primary yardstick of performance since it provides an overview of the possible cut-off levels in the test performance. The table shows that all the models are of high quality, as assessed by the training and test set statistics; binary descriptors tend to produce slightly better training models than VHSE descriptors. An increased number of neurons in the hidden layer improved the ability of our models to predict the intestinal permeability of peptide in the training set, however no such tendency was apparent in the statistics for the test set. This is presumably due to overtraining of the networks; as the network architecture becomes more complex, the number of parameters increases, entailing the risk of overtraining. The effect of overtraining was relatively small for the models based on VHSE descriptors, which use fewer variables than binary descriptors.

**Table 2:** Prediction accuracy for models with various network architectures^a^.

Binary Descriptor					VHSE Descriptor				
	
*N*_*hidden*_^b^	1 : 1 Data set		1 : 3 Data set		*N*_*hidden*_^b^	1 : 1 Data set		1 : 3 Data set	
			
	Training	Test	Training	Test		Training	Test	Training	Test
	
0	0.84	0.77	0.83	0.79	0	0.80	0.76	0.79	0.77
1	0.92	0.73	0.90	0.76	1	0.87	0.70	0.84	0.75
2	0.97	0.71	0.94	0.77	2	0.89	0.71	0.86	0.75
3	0.98	0.71	0.97	0.74	3	0.92	0.70	0.90	0.72

^a ^The network architecture A-B-C indicates the total number of descriptors in an input layer, where A is (7, the sequence length of a peptide) × (the number of descriptors for each amino acid), B and C are the numbers of neurons in hidden and output layers, respectively. For instance, the network architecture (7 × 20)-0-1 specifies a model constructed with zero neuron in hidden layer and one in output layer using the binary descriptor. All the models have one neuron in output layer.

^b ^The number(B) of neurons in a hidden layer.

To test the effect of the number of objects on overtraining, we also constructed neural network models for 1:3 data set in which the negative control data set was three times larger than the positive; Table 2 summarizes the capacity of these models for prediction. Considering models with the same network architectures, the differences in ROC scores between the training and test set were generally smaller in the 1:3 than the 1:1 data set. This result shows that the performance of the model is less affected by overtraining if the size of the data set is increased.

**Figure 2:**
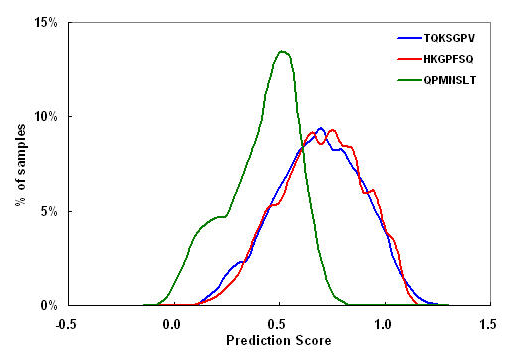
Distribution of prediction scores for all permutations of three peptide sequences.

To validate the sequence dependency, we compared the intestinal permeabilities of peptides with identical amino acid compositions but different sequences. We selected three peptides with different prediction scores from among the 852 intestinal barrier-permeable heptapeptides: TQKSGPV, with a high score (1.03), HKGPFQS, with a medium score (0.78), and QPMNSLT, with a low score (0.52). For each of these peptides, we generated a set of peptides with all the possible sequence permutations of the seven amino acids (7! = 5040) and intestinal permeabilities were predicted using the model with network architecture (7 × 20)-0-1. The wide distribution of the prediction scores for the peptide sets (Figure 2) clearly indicates that the intestinal permeability of a peptide depends on its sequence.

To evaluate the robustness of our models, we performed leave-5%-out cross-validation, which is analogous to leave-one-out cross-validation [28]: 5% of the sequences are omitted as validation data. The result of twenty rigorous tests (Table 3) shows only small differences between the different training runs, indicating that all the models are quite robust.

**Table 3:** The results of validation for models with network architecture (7 × 20)-0-1^a^.

Leave-5%-out cross-validation^b^		Decoy analysis^c^			
	
1 : 1 Data set		Real set		Decoy set	
	
Training^d^	Test^d^	Training	Test	Training	Test
0.841 ± 0.002	0.760 ± 0.005	0.82	0.74	0.70	0.47

^a ^The network architecture (7 × 20)-0-1 specifies a model constructed with zero neuron in hidden layer and one in output layer using the binary descriptor.

^b ^The results of rigorous test using leave-5%-out method in 1:1 data sets.

^c ^Comparison of ROC scores between real and decoy set using non-redundant data.

^d ^The results of 20 rigorous tests are averaged and expressed as mean ± standard deviation.

**Figure 3:**
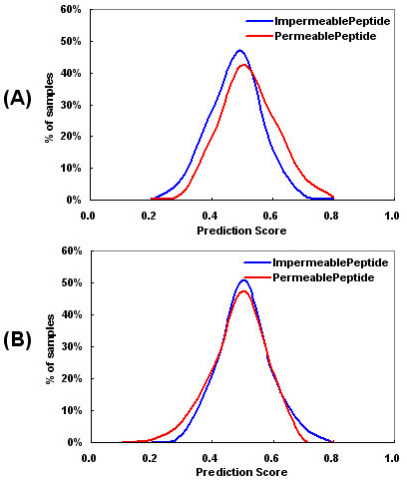
**The features of the model constructed with the decoy set**. The models were constructed with zero neuron in a hidden layer and one in an output layer using binary descriptor. (A) Training set and (B) Test set.

To test the reliability of the peptide sequences from the phage-display experiment as the positive control set and to validate the strength of our model in predicting the intestinal permeabilities of peptides, a separate decoy set was generated as positive control in the training and test set. We constructed supplementary model trained with the decoy set and compared that model with the model trained with the real data set for ability to discriminate between intestinal barrier-permeable and impermeable peptides. The predictive features presented in Figure 3 indicate that model constructed with the decoy set do not discriminate between these two permeability classes of peptides. Also, the validation result for model with network architecture (7 × 20)-0-1 (Table 3) suggest that the predictive power for the test set is considerably greater when the model is constructed with the real set than with the decoy. This result confirms that model trained with the real set is robust discriminator between intestinal barrier-permeable and impermeable peptides, and that the positive real data sequences were collected sufficiently well to allow efficient model construction.

More detailed statistics about the predictive capacities of our models are listed in Table 4, which shows a truth table analysis of the binary outcome based on intestinal permeability. The results show that our models are more sensitive in predicting the intestinal permeabilities of peptides for 1:1 than for 1:3 data set, while the specificity in screening out intestinal barrier-impermeable peptides is greater for 1:3 than for 1:1 data set. Thus, a neural network model based on 1:1 data set is preferred for selecting intestinal barrier-permeable peptides, while a model based on 1:3 data set is preferred for eliminating intestinal barrier-impermeable peptides. The accuracies of prediction by the models were strikingly high for both 1:1 and 1:3 data set.

**Table 4:** Comparison of truth table statistics for the test sets for two models

Network	1 : 1 Data set						1 : 3 Data set				
			
architecture	SE^a^	SP^b^	PPV^c^	NPV^d^	Acc^e^		SE^a^	SP^b^	PPV^c^	NPV^d^	Acc^e^
			
(7 × 20)-0-1	74	67	69	72	70		32	94	65	81	79
(7 × 8)-0-1	70	72	71	70	71		19	96	59	78	76

^a ^SE = Sensitivity : the proportion of all intestinal barrier-permeable peptides correctly predicted, SE = TP/(TP + FN) where TP is the number of intestinal barrier-permeable peptides correctly predicted and FN is the number of intestinal barrier-permeable peptides incorrectly predicted as impermeable peptides.

^b ^SP = Specificity : the proportion of intestinal barrier-impermeable peptides correctly predicted, SP = TN/(TN + FP) where TN is the number of intestinal barrier-impermeable peptides correctly predicted and FP is the number of intestinal barrier-impermeable peptides incorrectly predicted as permeable peptides.

^c ^PPV = Positive Predictive Value : the probability that a predicted permeable peptide is in fact a barrier- permeable peptide, PPV = TP/(TP + FP).

^d ^NPV = Negative Predictive Value : the probability that a predicted intestinal barrier-impermeable peptide is in fact impermeable peptide, NPV = TN/(TN + FN).

^e ^Acc = Accuracy : the percentage of all predictions that are correct, Acc = (TP + TN)/Total.

## Discussion

We have developed models for predicting the intestinal permeabilities of peptides. Our models produced nearly identical statistics for multiple training runs and efficiently discriminated among peptides on the basis of intestinal permeability. As shown in the decoy set analysis, models trained with random sequences had no prediction capacity, but the peptide sequences collected from the *in vivo *experiment served well as positive control sets for the QSAR models.

Although we tried to optimize the network architecture and to minimize overtraining and other related problems during the course of development, some factors in our model might cause prediction errors. We assumed that randomly-selected heptapeptide sequences can be used as negative controls. This assumption can be rationalized on the grounds that heptapeptides with random sequences are very likely to be intestinal barrier-impermeable because the sequences obtained from the *in vivo *experiment only covered a very small portion of the entire 'heptapeptide space'. Thus, our model correctly predicts permeable rather than impermeable peptides. This indicates that a model based on 1:3 data set is preferable for eliminating intestinal barrier-impermeable peptides if the random sequences chosen as negative controls do indeed show negligible intestinal absorption; as shown in Table 4, the specificity of 1:3 data set models is superior to that of 1:1 data set models. Consequently, our model has the best predictive power for the selection of the permeable peptide in relation to the reliability of the data set.

In this work, we developed models for prediction of intestinal permeabilities of peptides using the feed-forward neural network as an algorithm for training. As shown in Table 2, some problems could be detected in the feed-forward neural network, which are including overfitting, network architecture optimization, and selection of the best QSAR model. To avoid shortcomings such as overtraining which appears to have happened in our models with larger network complexity, robust QSAR models using the Bayesian regularized neural network would be more desirable [26,29]. The development of QSAR models using the more robust methods like the Bayesian neural network would be a fruitful approach of future work in terms of predictive ability and robustness of model for intestinal permeability of peptide.

Burden et al. [17] noted that property-based descriptors require a more flexible modeling method than binary descriptors to take account of larger contributions from cross terms or nonlinearity. However, our models produced very similar results on the discrimination of intestinal permeability using binary and VHSE descriptors; no statistically significant difference was observed in their ROC scores for the test sets.

The models most widely used for predicting passive intestinal absorption are drug-likeness prediction models such as the Rule of 5 model introduced by Lipinski et al. [30]; they have the advantages of being simple, easy to interpret and quick to compute. In general, such approaches are formulated on the basis of group additive methods, so the predicted intestinal permeability is similar for peptides consisting of the same numbers and types of amino acids, even though they may have different sequences. However, our analysis showed that the intestinal permeability of a peptide depends on its sequence (Figure 2) and cannot be explained simply by using the drug-likeness prediction models of passive transport. Because of its large size, the peptide-phage complex is expected to be transported across the intestinal barrier by other mechanisms such as transcytosis.

Systemic delivery of macromolecules via the oral pathway remains one of the most challenging problems in the drug delivery field, and transcytosis may be a mechanism for transporting therapeutic agents across the intestinal barrier. If a carrier molecule, either a natural ligand or an antibody binding to a transcytotic receptor on the intestinal epithelium, is covalently bound to a therapeutic agent by a short linker, the conjugate can bind to the cognate receptor and undergo vesicular trafficking across the intestinal barrier [31]. This 'carrier-drug conjugate' approach has been tried using drugs conjugated to immunoglobulin G (IgG), lactoferrin, transferrin or folic acid, all of which have cognate transcytosis receptors in enterocytes [32]. To utilize the transcytosis mechanism for an oral drug delivery system, it is essential to identify ligands that can bind to the receptors and facilitate efficient transcytosis across the intestinal barrier. Our QSAR study on the selection of intestinal barrier-permeable peptides should be applicable to the development of peptide 'carriers' for delivering large molecules such as proteins and drugs.

## Conclusion

We used artificial neural networks to develop the first models for predicting the intestinal permeabilities of peptides on the basis of sequence information. The high quality models obtained were capable of making reliable predictions. These models are expected to find applications in the selection of intestinal barrier-permeable peptides from large peptide libraries, and the selected peptides might be used to facilitate the transport of large molecules across the intestinal barrier.

## Methods

### Preparation of intestinal barrier-permeable peptides

**Figure 4:**
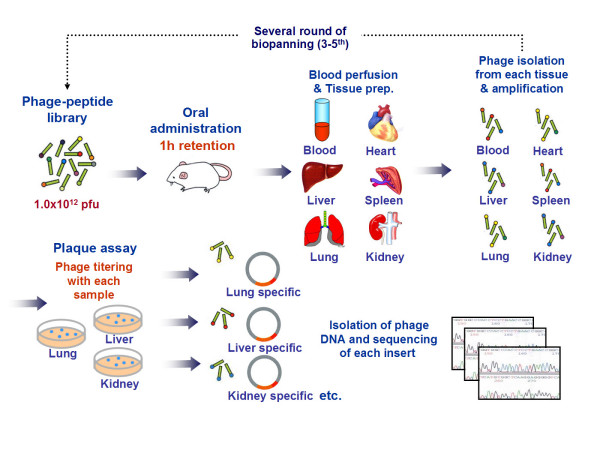
**A schematic view of peroral phage display procedure**. After the third round of biopanning, individual recombinant phage was randomly selected from each organ tissue elute for analysis of peptide sequences from their genomes.

To identify peptides transported across the intestinal barrier, an *in vivo *phage display technique was developed using the disulfide-constrained cyclic M13 phage display library. This displays random 7 amino acids with 2 flanking cysteines at both ends of the peptide at the N-terminus of the M13 phage pIII protein (Ph.D.-C7C system: complexity = 1.2 × 10^9^, New England BioLabs, Beverly, Maryland). For the first round of biopanning (*in vivo *selection), 1.2 × 10^12 ^pfu (approximately 1,000 copies for each peptide-coding phage recombinant) of the Ph.D.-C7C library in 500 μl phosphate-buffered saline (PBS) was administered orally to four overnight-starved adult Sprague-Dawley rats (12 weeks old, male; Samtako, Osan, Korea). One hour after oral administration of the phage library, the rats were sacrificed by an abdominal incision under deep anesthesia (ketamine hydrochloride, 80 mg/kg bw; xylazine, 10 mg/kg bw) and perfused via the left ventricle with 120 ml heparin-supplemented DMEM (Dulbecco's Modified Eagle's Media, GIBCO, USA) to ensure phage clearance from the blood pool. Representative inner organs (liver, lung, heart, spleen and kidney) were extracted, roughly chopped separately on a Petri dish and washed three times with 30 ml ice-cooled PBS. Each drained organ sample was resuspended in 2 ml TSS [Tissue Suspension Solution: DMEM, 1% (w/v) BSA (bovine serum albumin), 10% (v/v) protease inhibitor cocktail (Sigma, USA)] in a separate 50 ml polycarbonate tube, then homogenized. The phage transported into each organ across the intestinal barrier were eluted from each homogenized organ tissue sample by vigorous vortexing with 2 ml 0.1 M glycine, pH 2.0, and centrifugation at 14,000 g for 8 min. Each supernatant was neutralized with 55–60 μl 2 M Tris base. The phage eluted from each organ sample were quantified by titration, suspended in the same volume (100 μl) and then amplified by infection of an *Escherichia coli *host ER 2738 (New England BioLabs) for the next round of biopanning. The amplified phage samples were concentrated using 3.3% polyethylene glycol 8,000/0.4 M NaCl (Sigma, USA) and quantified again by titration. The second round of biopanning was initiated by oral administration of the newly amplified phage (1.2 × 10^12 ^pfu) to rats (n = 4). The sequential procedure described above was named the 'peroral phage display' (Figure 4). After a third round of biopanning, individual recombinant phage was randomly selected from each organ eluate for the analysis of peptide sequences.

### Data sets

The positive control data set of peptides that can cross the intestinal barrier was obtained from the 852 heptapeptide sequences identified by the peroral phage display experiments. The negative control data set was generated from random sequences that had the same frequencies of occurrence of each amino acid residue as in the Ph.D.-C7C phage library. The random sequences were then compared with the positive control data and any common sequences were removed from the negative control data. For 1:1 data sets, the positive and negative control data comprised the same number of peptides. To evaluate the effect of data size, we also generated second data set in which there was three times more negative control than positive control data. The 2556 random heptapeptide sequences were used as the negative control in 1:3 data set. About 80% of each data set was used for network training and the remaining data were used for the test set to validate the trained network.

### Descriptors

Two types of amino acid descriptors, binary and VHSE, were used to encode important features of individual peptide sequences. The binary descriptor used a set of 20 binary digits to encode each amino acid (all zeros except for the one characterizing the given amino acid) [17]. For example, 7 × 20 = 140 variables were used to encode a heptapeptide. The VHSE descriptor is a property descriptor composed of 8 variables for each amino acid and characterizes the hydrophobic, steric and electronic properties of the 20 coded amino acids [33]. For a heptapeptide, 7 × 8 = 56 variables were used to build models based on this descriptor.

### Neural network model

We used the machine-learning method to drive structure-activity relationships. The calculations were carried out on a Pentium 2.2 GHz machine using the nnet of the VR 7.2 package [34] for feed-forward neural networks with a single hidden layer and for multinomial log-linear models. We used a three-layer neural network architecture containing a single hidden layer in which the number of neurons was increased from 0 to 3. This network consisted of a multilayer system of neurons, with each neuron in a given layer fully connected to all the neurons in the two adjacent levels. A neural network was trained to map a set of input data to a corresponding set of output data by iterative adjustment of the weights. The activation function of the hidden layer units is the logistic function and the output units are linear. The Broyden-Fletcher-Goldfarb-Shanno (BFGS) method was used as the optimization function. To help the optimization process and to avoid over-fitting, the weight decay was set at 0.001. The maximum number of iterations for network training was 50,000 and the other parameters were given the default values set by the nnet of the VR 7.2 package. Before the learning network was applied, the input value of the positive control was 0.9 and that of the negative control was 0.1.

### Evaluation

To score the models, the ROC score, which is the area under the ROC curve [35], was used for each training and test set. The score is 1 for a perfect classification and 0.5 for a random classification. All the ROC scores reported were generated from a leave-group-out cross-validation of real and decoy set.

### Validation using decoy set

We prepared supplementary model trained with decoy set [36] and compared that model with the model trained with real data set for the ability to discriminate between intestinal barrier-permeable and impermeable peptides. The positive as well as the negative control data of the decoy set was generated from random peptides. The decoy set was prepared carefully to ensure that there was no redundant peptide in the positive control data and no overlap between the positive and the negative control data. To ensure the consistency of data, non-redundant peptide subset of the real set was also prepared; the positive control data set prepared comprised 677 peptides.

## Authors' contributions

EJ, MK, KC and JMS participated in the design of the neural network architecture. EJ, JK, HR and DHJ built, trained and tested the neural networks. SKK and MKK collected sequence data from experimental study and CHY participated in the design of the experimental study. EJ, JK and SKK authored the manuscript. DHJ, KC, JMS and SHC revised the manuscript. YJC and SHC supervised this work. All authors read and approved the final manuscript.
